# Plant Molecular Farming for Cancer Immunotherapy: Production, Glycoengineering, and Structural Insights into Therapeutic Monoclonal Antibodies

**DOI:** 10.3390/cimb48101028

**Published:** 2026-10-04

**Authors:** Maria Concepción Tamayo-Ordoñez, María José García-Castillo, Francisco Alberto Tamayo-Ordoñez, Víctor de Jesús Suárez-Valencia, Atl Víctor Córdova-Quiroz, Víctor Hugo Ramos-García, Benjamín Abraham Ayil-Gutiérrez, Angel Virgilio Domínguez-May, Sara Luz Nahuat-Dzib, Juan Carlos Robles-Heredia, Marta Guadalupe Lizama-Gasca, Daniel Lozano-Venegas, Víctor Manuel Velasco-Rodríguez, Esmeralda Cazares-Sánchez, Grethel Díaz-Palafox, Yahaira de Jesús Tamayo-Ordoñez

**Affiliations:** 1 Facultad de Ciencias Químicas, Universidad Autónoma de Coahuila, Saltillo 25280, Mexico; mtamayo@uadec.edu.mx; 2 Unidad de Biotecnología, Centro de Investigación Científica de Yucatán A.C., Mérida 97200, Mexico; mjgc.2589@gmail.com (M.J.G.-C.); mar_10914@hotmail.com (M.G.L.-G.); 3 Facultad de Química, Universidad Autónoma del Carmen, Ciudad del Carmen 24180, Mexico; ftamayo@pampano.unacar.mx (F.A.T.-O.); jrobles@pampano.unacar.mx (J.C.R.-H.); 4 Facultad de Medicina, Universidad Autónoma de Coahuila, Saltillo 25280, Mexico; victorsuarezvalencia@uadec.edu.mx; 5 Unidad Académica Multidisciplinaria Reynosa-Aztlán, Universidad Autónoma de Tamaulipas, Reynosa 88740, Mexico; vramosg1600@gmail.com; 6 SECIHTI, Centro de Biotecnología Genómica, Instituto Politécnico Nacional, Reynosa 88710, Mexico; bayil@ipn.mx; 7 TecNM-Instituto Tecnológico Superior del Sur del Estado de Yucatán, Oxkutzcab 97880, Mexico; adominguez@suryucatan.tecnm.mx; 8 Departamento de Ingeniería Química y Bioquímica, Campus Mérida, Tecnológico Nacional de México, Mérida 97118, Mexico; sara.nd@merida.tecnm.mx; 9 Facultad de Enfermería y Nutrición U.L., Universidad Autónoma de Coahuila, Torreón 27298, Mexico; lozano.daniel@uadec.edu.mx (D.L.-V.); victorvelasco@uadec.edu.mx (V.M.V.-R.); 10 Instituto Tecnológico de la Zona Maya, Tecnológico Nacional de México, Chetumal 77960, Mexico; esmecs_13@hotmail.com

**Keywords:** plant molecular farming, cancer immunotherapy, monoclonal antibodies, glycoengineering

## Abstract

Cancer is one of the leading causes of mortality worldwide, creating an urgent need for innovative, scalable, and cost-effective therapeutic strategies. Monoclonal antibodies have transformed modern oncology by enabling highly specific targeting of tumor-associated antigens and immune checkpoint pathways. However, their production in conventional mammalian cell systems remains expensive and requires extensive infrastructure. Plant molecular bioengineering has emerged as a promising alternative platform for the production of recombinant biopharmaceuticals, offering advantages such as rapid scalability, reduced manufacturing costs, biosafety, and flexible expression systems. This review summarizes recent developments in the production of plant-based anticancer biologics, with particular emphasis on therapeutic monoclonal antibodies and immune checkpoint inhibitors. The review includes plant-derived antibodies targeting HER2, PD-1, and PD-L1, which have demonstrated structural integrity, antigen-binding capacity, and biological activity comparable to those of their mammalian-derived counterparts. In this study, we performed a comparative computational structural analysis of trastuzumab and pembrolizumab using experimentally determined antibody structures as references and computationally modeled plant-associated Fc glycoforms, with particular emphasis on Fc N-glycosylation and glycan solvent accessibility. Overall, advances in synthetic biology, precision glycoengineering, and controlled cultivation technologies highlight the potential of plant biofactories as promising platforms for the development of next-generation, affordable, and globally accessible cancer immunotherapies.

## 1. Introduction

Cancer is one of the leading causes of mortality worldwide and represents a major public health challenge due to its increasing incidence, high treatment costs, therapeutic resistance, and growing socioeconomic burden. Recent advances in precision medicine, immunotherapy, and targeted therapies have significantly improved patient survival and quality of life. However, the large-scale production of biopharmaceuticals, including monoclonal antibodies, recombinant proteins, cytokines, and therapeutic vaccines, remains constrained by high manufacturing costs, complex purification procedures, and stringent regulatory requirements, which may limit global access to advanced therapies [1].

Against this backdrop, molecular agriculture has emerged as an innovative and sustainable strategy for producing recombinant biomolecules using plant-based production systems, often referred to as biofactories. Compared with conventional microbial and mammalian cell culture systems, plant-based expression platforms offer several advantages, including lower production costs, reduced infrastructure requirements, rapid scalability, and a minimal risk of contamination with human pathogens. In addition, plants possess the cellular machinery required for the synthesis, assembly, and post-translational modification of complex recombinant proteins, making them attractive hosts for the production of therapeutic antibodies and other biopharmaceuticals [1,2].

Among plant-based production platforms, *Nicotiana benthamiana* has become one of the most widely used hosts because of its compatibility with *Agrobacterium*-mediated transient expression systems, which enable the rapid production of recombinant proteins. Rice, corn, and lettuce have also shown potential as alternative production platforms for vaccines, antibodies, enzymes, and other therapeutic proteins. In addition, advances in synthetic biology, transient expression technologies, glycoengineering, and genome editing have improved recombinant protein yields and product quality, thereby accelerating the development of plant molecular farming systems [2,3,4].

Interest in the development of plant-based pharmaceuticals has increased following the successful commercialization and clinical evaluation of plant-derived recombinant vaccines, enzymes, and therapeutic proteins. Recent technological and economic analyses indicate that plant molecular farming can significantly reduce manufacturing costs while maintaining product quality and scalability. These characteristics are particularly important for improving access to biologic therapies in low- and middle-income countries, where the high cost of monoclonal antibody treatments remains a major barrier to equitable healthcare access [1,3].

Through plant molecular bioengineering, therapeutic monoclonal antibodies for cancer treatment are already being produced. Monoclonal antibodies targeting clinically relevant biomarkers, such as HER2, PD-1, PD-L1, and VEGF, have transformed modern oncology by enabling highly targeted and effective therapeutic interventions. Recent advances in plant glycoengineering have further strengthened the potential of plant-based expression systems by enabling the generation of humanized glycosylation profiles that more closely resemble those produced in mammalian cells, thereby improving product comparability and potentially enhancing therapeutic performance [4,5].

In this review, we examine recent advances in plant molecular bioengineering for cancer therapy, with particular emphasis on the production of therapeutic monoclonal antibodies, glycoengineering strategies, structural characterization, and emerging applications in oncology. Particular attention is given to plant-derived trastuzumab and pembrolizumab as representative case studies that highlight the potential of plant biofactories for the future development of affordable and scalable cancer immunotherapies.

## 2. Current Approaches of Molecular Farming in Cancer Therapy

### 2.1. General Workflow of Plant Molecular Farming for Anticancer Antibody Production

Over the past decade, advances in plant molecular farming have transformed plants into efficient biofactories capable of producing complex therapeutic proteins for applications in oncology, infectious diseases, and personalized medicine [1,2]. Although the production of recombinant proteins in plants is now well established, several critical factors determine the successful production of the recombinant protein of interest, particularly therapeutic antibodies. Figure 1 provides a general overview of the production of therapeutic drugs using plant-based expression systems. First, target genes with biological functions relevant to cancer cell destruction and/or inhibition of proliferation must be identified. Codon optimization and vector engineering are then commonly employed to maximize recombinant protein expression. The optimized genes are subsequently cloned into plant expression vectors and introduced into *Agrobacterium tumefaciens*, which is used to transform *Nicotiana benthamiana*. Following agroinfiltration, recombinant antibodies accumulate in leaf tissues over several days, enabling rapid protein production compared with conventional mammalian cell culture systems [2,6].

An important and emerging aspect of glycoengineering involves modifying endogenous plant glycosylation pathways to generate N-glycan structures that are more compatible with human glycosylation profiles. Native plant glycans differ from mammalian glycans because they contain β1,2-xylose and α1,3-fucose residues in the core of N-glycan structures, which may affect antibody immunogenicity and Fc-mediated effector functions. The development of glycoengineered plant lines that lack plant-specific glycosyltransferases or express mammalian glycosylation enzymes has become an important strategy for increasing the comparability of plant-derived antibodies with their mammalian-produced counterparts and for modulating their functional properties [4,5,7].

Recombinant proteins are extracted from plant tissues and subsequently purified using methods such as affinity chromatography and ion-exchange chromatography, together with additional purification steps designed to achieve the required pharmaceutical purity. The choice of purification strategy depends on the characteristics of the recombinant protein and the expression system used for its production. Subsequently, comprehensive quality control analyses are performed to assess protein integrity, aggregation status, glycosylation profiles, antigen-binding affinity, and Fc-mediated biological activity. Analytical techniques such as mass spectrometry, high-performance liquid chromatography (HPLC), capillary electrophoresis, and structural modeling can be used to evaluate these parameters and ensure compliance with regulatory requirements and product consistency [1,3,8].

Together, these integrated technologies have transformed plant molecular farming into a robust platform for the production of recombinant monoclonal antibodies for applications in cancer immunotherapy. The combination of rapid transient expression systems, advanced glycoengineering strategies, and sophisticated analytical characterization enables the generation of plant-derived therapeutic antibodies with properties that are increasingly comparable to those of antibodies produced in conventional mammalian expression systems [2,4].

### 2.2. Successful Plant-Derived Therapeutic Antibodies for Cancer Therapy

Monoclonal antibodies (mAbs) represent one of the most successful classes of biopharmaceuticals in modern oncology because of their high specificity for tumor-associated antigens and their ability to modulate immune responses against cancer cells. Therapeutic antibodies targeting molecules such as human epidermal growth factor receptor 2 (HER2), programmed cell death protein 1 (PD-1), programmed death ligand 1 (PD-L1), and vascular endothelial growth factor (VEGF) have significantly improved clinical outcomes across a wide range of malignancies, including breast, lung, and colorectal cancers, as well as hematological malignancies [9]. Despite their clinical success, the high production costs, sophisticated infrastructure, and complex requirements associated with their manufacture, application, and storage can contribute to high treatment costs and limit access to these therapies, particularly in low- and middle-income countries [1,2]. Conventional mammalian expression systems, such as Chinese hamster ovary (CHO) cells, are widely used for mAb production but require substantial infrastructure and stringent manufacturing conditions.

With the aim of reducing the costs associated with cancer therapies, plant molecular bioengineering has emerged as a promising alternative platform for the production of recombinant monoclonal antibodies for potential therapeutic applications. *Nicotiana benthamiana* has demonstrated remarkable efficiency in the transient expression of fully assembled and functional antibodies with therapeutic potential. These systems offer several advantages, including rapid biomass generation, reduced production costs, scalability, and a lower risk of contamination with human pathogens [1,2].

Recent work has further expanded the application of plant-based expression systems to immune checkpoint targets. Moon et al. demonstrated the production of recombinant human PD-1 in *Nicotiana benthamiana* and showed that the plant-derived antigen elicited antibodies capable of recognizing native PD-1 on the cell surface, supporting the potential of plant-produced antigens for therapeutic antibody discovery [10]. In a complementary approach, Rattanapisit et al. produced the anti-PD-L1 monoclonal antibody atezolizumab in *N. benthamiana* and evaluated its immunotherapeutic potential in vitro and in vivo [11].

Recombinant atezolizumab, an anti-PD-L1 monoclonal antibody transiently expressed in *Nicotiana benthamiana*, exhibited high binding affinity for PD-L1 and showed antitumor activity comparable to that of the commercially available therapeutic antibody Tecentriq^®^ (Genentech, South San Francisco, CA, USA). These findings support the feasibility of plant expression systems for the production of complex immune checkpoint inhibitors and suggest their potential to reduce manufacturing costs and improve access to advanced cancer therapies [11].

Another example is Avaren-Fc (AvFc), a glycosylation-modified lectin–Fc fusion protein produced in *Nicotiana benthamiana*. AvFc selectively recognizes cancer-associated high-mannose glycans, which are frequently overexpressed on malignant cells. Glycoengineering strategies enhanced Fc receptor interactions and antitumor activity, demonstrating that plant molecular farming can support not only the production of conventional monoclonal antibodies but also the development of next-generation engineered biologics with novel anticancer mechanisms [12].

It is important to note that the development of glycoengineered plant lines capable of producing humanized N-glycan structures has addressed one of the major limitations historically associated with plant-derived biopharmaceuticals. Glycoengineering of plant expression systems can improve interactions with Fc receptors, reduce potential immunogenicity, and increase the structural and functional comparability of plant-derived antibodies with clinically approved mammalian-produced antibodies [4,5,7].

In addition, evidence is emerging for the plant-based production of antibody fragments targeting angiogenesis-related pathways, particularly the VEGF/VEGFR axis. VEGF signaling through VEGFR2 plays a central role in tumor angiogenesis and represents an important molecular target for antiangiogenic cancer therapies. Notably, an anti-VEGFR2 nanobody has been transiently expressed in *Nicotiana benthamiana* and *N. tabacum* using a plant viral vector, demonstrating the feasibility of producing angiogenesis-targeting antibody fragments in plant expression systems [13]. These findings expand the potential applications of plant molecular farming beyond conventional full-length monoclonal antibodies toward smaller antibody-derived molecules targeting pathways involved in tumor vascularization.

Plant bioengineering has also enabled the development of virus-derived nanoparticles as modular platforms for targeted biomedical applications. Recently, genetically encoded potato virus X (PVX)-derived nanoparticles displaying nanobodies against the cancer-associated receptors EGFR and HER2 were successfully produced in *Nicotiana benthamiana* [14]. The recombinant nanoparticles showed successful self-assembly and structural integrity, while immunoelectron microscopy confirmed the surface display of the corresponding nanobodies. Functional characterization by flow cytometry and confocal microscopy further demonstrated binding of the nanobody-decorated PVX nanoparticles to tumor cells expressing their respective EGFR or HER2 targets [14]. Moreover, selected HER2-targeted PVX nanoparticles were evaluated in tumor-bearing mice, where nanobody decoration was associated with increased tumor retention and recruitment of myeloid cells within the tumor microenvironment [14]. These findings provide experimental support for the use of plant-produced viral nanoparticles as adaptable scaffolds for cancer targeting and immunotherapy research.

More broadly, the successful plant-based production and functional presentation of nanobodies directed against clinically relevant tumor-associated receptors, including VEGFR2, HER2, and EGFR, illustrates the versatility of plant expression systems for generating next-generation antibody-derived biologics [13,14]. Taken together, these studies highlight the expanding role of plant bioengineering in the development of monoclonal antibody-, nanobody-, and viral nanoparticle-based strategies with potential applications in cancer therapy.

### 2.3. Principal Glycosylation Differences Between Mammalian- and Plant-Derived Antibodies

Trastuzumab is a monoclonal antibody widely used for the treatment of HER2-positive breast and gastric cancers and represents an important model for evaluating the impact of glycosylation on antibody functionality. HER2 overexpression occurs in approximately 15–20% of breast cancer cases and is associated with aggressive tumor progression and poor clinical prognosis [15,16]. Trastuzumab exerts its therapeutic effects by binding to the extracellular domain of HER2, thereby inhibiting receptor signaling and promoting antibody-dependent cellular cytotoxicity (ADCC) through interactions between its Fc region and Fcγ receptors expressed on immune effector cells [17].

The biological activity of therapeutic antibodies is strongly influenced by Fc-associated N-glycosylation, one of the most important post-translational modifications affecting protein stability, immunogenicity, and effector functions. In IgG1 antibodies such as trastuzumab, N-glycosylation occurs predominantly at the conserved Asn297 residue within the Fc domain. Variations in glycan composition can significantly affect the binding affinity of antibodies for Fcγ receptors and consequently modulate ADCC activity and therapeutic efficacy [18].

Commercial trastuzumab formulations, including Herceptin^®^ and several approved biosimilars, are predominantly produced in Chinese hamster ovary (CHO) cells because these systems generate glycosylation profiles that are highly compatible with human immune functions [19]. CHO-derived antibodies typically contain complex, biantennary N-glycans characterized by core α(1,6)-fucosylation, terminal galactose residues, and variable levels of sialylation. These glycan features contribute to the well-characterized pharmacokinetic and pharmacodynamic properties of clinically approved therapeutic antibodies [18,19].

In contrast, plant expression systems such as *Nicotiana benthamiana* naturally synthesize N-glycans containing β(1,2)-xylose and α(1,3)-fucose residues, which are not typically present in mammalian-derived N-glycans. These plant-specific glycan motifs may influence Fc receptor interactions and have historically raised concerns regarding potential immunogenicity and altered therapeutic properties [4,7,20]. Nevertheless, numerous studies have demonstrated that plant-produced antibodies can retain correct assembly, antigen-binding capacity, and biological activity despite these structural differences [10,11].

Plant glycoengineering through the development of *N. benthamiana* lines lacking the activities of β1,2-xylosyltransferase and α1,3-fucosyltransferase (ΔXT/FT plants) has enabled the production of recombinant antibodies with more human-like N-glycan profiles. These glycoengineered plants can reduce the incorporation of plant-specific glycans and, in some antibody models, enhance binding to FcγRIIIa, thereby increasing ADCC activity and potentially improving the functional properties of the resulting antibodies [5,20,21].

Recent advances in synthetic biology have further enabled the introduction of mammalian glycosylation pathways into plant hosts, including galactosylation and terminal sialylation modules. These strategies represent an important step toward generating plant-produced antibodies with glycosylation profiles that more closely resemble those of commercially produced biopharmaceuticals, thereby improving molecular comparability and potentially facilitating their development for therapeutic applications [21,22]. Table 1 summarizes selected trastuzumab-based biopharmaceuticals produced in mammalian and plant expression systems and highlights variations in their glycosylation characteristics. Figure 2 summarizes the major differences in glycosylation between mammalian-derived and plant-derived trastuzumab and illustrates the role of glycoengineering in the generation of plant-produced antibodies with more human-like glycosylation profiles.

### 2.4. Trastuzumab and Pembrolizumab as a Case Study of Plant-Derived Cancer Immunotherapy

Recent experimental studies further support the feasibility of plant-based platforms for the production and characterization of therapeutic monoclonal antibodies. Advances in plant glycoengineering have demonstrated the potential to generate antibodies with modified Fc N-glycosylation profiles and altered functional properties [25]. More specifically, glycoengineered rice cell-based systems have been used to produce trastuzumab with modified N-glycosylation and enhanced functional properties [26]. In parallel, recombinant plant-produced pembrolizumab has been subjected to detailed analytical characterization, including LC-MS-based assessment of *Nicotiana benthamiana* host plant proteins in the final drug product, highlighting the importance of product-specific quality control in plant-based antibody manufacturing [27].

#### 2.4.1. Structural Modeling and Comparative Fc Glycosylation Analysis

To investigate the structural consequences associated with differences in Fc N-glycosylation between mammalian and plant expression systems, a comparative in silico analysis was performed using trastuzumab (IgG1) and pembrolizumab (IgG4) as representative therapeutic monoclonal antibodies. Experimentally determined structures deposited in the Protein Data Bank (PDB) were used as the starting structural references, whereas plant-associated glycoforms were generated computationally by remodeling the Fc N-glycans while preserving the corresponding experimental protein scaffold.

For trastuzumab, two experimentally determined structures were considered according to the structural region being analyzed. The trastuzumab Fab–HER2 complex (PDB ID: 6OGE) was used exclusively as a reference for the antigen-binding region because this structure contains the Fab fragment but does not include the Fc domain. Consequently, PDB 6OGE was not used for Fc glycosylation modeling or quantitative Fc analysis. Instead, the experimentally determined trastuzumab Fc structure deposited under PDB ID 9Z0F was used as the structural reference for the Fc region. This structure contains the Fc dimer and experimentally resolved Fc-associated N-glycans and was therefore used as the starting structure for glycan remodeling and quantitative structural analysis. Non-antibody components not required for the analysis, including crystallographic water molecules and the linker-payload associated with the deposited Fc construct, were excluded prior to quantitative analysis. The experimentally resolved Fc glycans in 9Z0F, displaying a G0F-like composition, were retained in their experimental structural environment and used as the mammalian reference rather than replacing them with idealized human-type glycan structures.

For pembrolizumab, the experimentally determined full-length IgG4 structure deposited under PDB ID 5DK3 was used as the primary structural reference. Unlike the Fab-containing pembrolizumab–PD-1 complex deposited under PDB ID 5GGS, 5DK3 contains the complete IgG4 antibody and therefore provides structural information for both the Fab and Fc regions. PDB 5GGS was consequently not used for Fc modeling or quantitative glycan analysis. In 5DK3, the conserved Fc N-glycosylation sites correspond to Asn297 of heavy chains B and G. The experimentally resolved N-glycans associated with these sites were retained as the mammalian reference for quantitative comparison. Importantly, the two resolved Fc glycans in 5DK3 were analyzed individually because they are structurally asymmetric: the glycan associated with Asn297 of heavy chain B (glycan chain C) lacks a resolved core fucose, whereas the glycan associated with Asn297 of heavy chain G (glycan chain D) contains a resolved fucose residue. Thus, the experimentally determined glycan structures were analyzed directly rather than assuming two identical mammalian Fc glycans.

Plant-associated Fc glycoforms of both antibodies were generated using the GLYCAM-Web Glycoprotein Builder. For trastuzumab, plant-associated glycans were introduced at Asn297 of Fc chains A and B using the 9Z0F protein coordinates as the structural scaffold. For pembrolizumab, the corresponding glycans were introduced at Asn297 of heavy chains B and G using the 5DK3 coordinates. In both antibodies, the same representative plant complex N-glycan, GnGnXF, was used to ensure methodological consistency between the two comparative analyses. This glycan contains the characteristic plant-associated β1,2-xylose and core α1,3-fucose [28] residues and was defined in GLYCAM using the condensed sequence DGlcpNAcb1-2DManpa1-6[DGlcpNAcb1-2DManpa1-3][DXylpb1-2]DManpb1-4DGlcpNAcb1-4[LFucpa1-3]DGlcpNAcb1-OH.

The same GLYCAM-Web modeling parameters were applied to trastuzumab and pembrolizumab. Glycoprotein structures were generated using an overlap rejection threshold of 2.0 Å, a search exhaustiveness value of 5, and a random seed of 12,345. No additional glycan conformations were requested. The *Prepare for MD* option was enabled, whereas *Retain Initial Glycosite Orientation*, *Delete Unresolvable Glycans*, and *Move Overlapping Protein Sidechains* were disabled. The resulting GnGnXF-containing structures were considered representative static computational glycoforms of plant-associated antibodies and were not interpreted as experimentally determined structures of plant-produced trastuzumab or pembrolizumab.

Structural comparison between each experimentally determined mammalian reference and its corresponding plant-associated model was performed by least-squares superposition of equivalent Fc Cα atoms. For trastuzumab, corresponding Cα atoms from Fc chains A and B of the 9Z0F reference and plant-associated model were used, whereas the corresponding Fc portions of heavy chains B and G were used for pembrolizumab. Root-mean-square deviation (RMSD) was calculated from the aligned Fc Cα coordinates to assess whether the protein backbone was altered during computational glycan remodeling. Because the plant-associated models were constructed directly from the experimental protein coordinates, these RMSD values were interpreted primarily as indicators of preservation of the starting Fc scaffold during modeling rather than as measurements of glycan-induced conformational dynamics.

Differences in Fc glycan exposure were quantitatively evaluated by calculating the solvent-accessible surface area (SASA) of each N-glycan. SASA calculations were performed using the Shrake–Rupley algorithm with a solvent probe radius of 1.4 Å and 960 surface points per atom. Importantly, the glycans were not analyzed as isolated carbohydrate structures. Instead, the solvent-accessible area of the atoms belonging to each Fc-associated glycan was calculated while retaining the surrounding Fc protein, thereby accounting for steric shielding and the local structural environment of the glycosylation site. Individual glycan SASA values were determined for each Fc glycosylation site, followed by calculation of the mean SASA for each mammalian reference and its corresponding plant-associated model. Absolute (ΔSASA) and relative percentage changes in mean glycan solvent accessibility were subsequently calculated for comparison between mammalian and plant-associated glycoforms.

Molecular inspection, structural representations, and visualization of Fc glycosylation sites were performed using PyMOL Molecular Graphics System v2.5 and UCSF ChimeraX v1.6. These programs were used to inspect the experimental structures, visualize the Fc-associated glycans and Asn297 glycosylation sites, and represent the structural superpositions used in Figure 3 and Figure 4. GLYCAM-Web Glycoprotein Builder was specifically used for construction of the plant-associated GnGnXF glycoforms, whereas the experimentally resolved glycans present in PDB 9Z0F and 5DK3 were used directly as the mammalian structural references. Thus, the glycan modeling and visualization steps were clearly separated from the quantitative RMSD and SASA analyses.

All comparisons represent static structure-based computational analyses. Accordingly, differences in glycan composition, conformation, or calculated solvent accessibility were not interpreted as direct evidence of changes in Fc receptor binding, effector functions, pharmacokinetics, immunogenicity, or in vivo biological activity [29]. Rather, the models were used to evaluate structural differences associated with representative mammalian and plant-associated Fc glycosylation patterns and to provide a reproducible structural framework for the comparative analyses presented in Figure 3 and Figure 4.

#### 2.4.2. Comparative Structural Analysis of Mammalian- and Plant-Derived Trastuzumab

Figure 3 presents a comparative structural analysis of the experimentally determined mammalian trastuzumab Fc reference and a computationally modeled plant-associated Fc glycoform. The analysis focused on the conserved N-glycosylation site at Asn297 and evaluated differences in glycan composition, structural organization, and solvent accessibility.

Panel A shows the mammalian trastuzumab reference based on the experimentally determined Fc structure deposited under PDB ID 9Z0F. The Fc-associated N-glycans resolved in this structure displayed a G0F-like composition and were retained in their experimentally determined structural environment. These glycans therefore served as the mammalian reference for subsequent structural and solvent-accessibility comparisons rather than being reconstructed as hypothetical human-like glycoforms.

Panel B shows the plant-associated trastuzumab model generated from the same 9Z0F Fc coordinates using GLYCAM-Web Glycoprotein Builder. The experimentally resolved mammalian glycans were replaced at Asn297 of chains A and B with representative plant-associated GnGnXF N-glycans containing the characteristic β1,2-xylose and core α1,3-fucose residues. This approach allowed the influence of glycan composition and spatial organization to be examined while maintaining the same Fc protein scaffold as the structural reference.

Panel C provides a comparative surface representation of the Fc glycosylation environment in the mammalian and plant-associated models. Differences are apparent in the spatial distribution of the Fc-associated glycans as a consequence of their distinct carbohydrate compositions. This representation was used primarily to visualize the molecular environment surrounding the glycosylation sites and should not be interpreted as direct evidence of altered Fc receptor binding or biological activity.

Panel D shows the structural superposition of the mammalian reference and plant-associated Fc models. Least-squares superposition of corresponding Fc Cα atoms resulted in an RMSD of 0.002 Å, demonstrating that the Fc protein backbone was essentially preserved during glycan remodeling. Thus, the introduction of the GnGnXF glycans did not produce an appreciable change in the starting Fc backbone coordinates in the static computational model. Because the plant-associated structure was generated by remodeling glycans on the 9Z0F Fc scaffold, this very low RMSD primarily reflects preservation of the starting protein coordinates and should not be interpreted as evidence that plant-associated glycosylation cannot influence Fc conformational dynamics under physiological conditions.

Panel E quantitatively compares the solvent-accessible surface area (SASA) of the Fc-associated N-glycans. In the mammalian reference, glycan SASA values of 1063.00 Å^2^ and 1029.44 Å^2^ were obtained for chains A and B, respectively, resulting in a mean SASA of 1046.22 Å^2^. In contrast, the modeled plant-associated GnGnXF glycans displayed SASA values of 1624.65 Å^2^ and 1518.09 Å^2^ for chains A and B, respectively, with a mean value of 1571.37 Å^2^. The plant-associated model therefore exhibited an increase in mean glycan SASA of 525.14 Å^2^, corresponding to approximately 50.19% greater solvent accessibility relative to the mammalian reference.

Overall, the comparative analysis showed that remodeling the Fc-associated glycans from the experimentally resolved mammalian G0F-like structures to plant-associated GnGnXF structures preserved the Fc protein backbone while substantially altering the calculated solvent exposure of the N-glycans. The approximately 50% increase in mean glycan SASA observed in the plant-associated model indicates a marked difference in the molecular accessibility of the Fc glycan region without a corresponding alteration of the static Fc backbone. These findings provide a structural basis for considering glycan composition and accessibility in the glycoengineering of plant-produced therapeutic antibodies. However, because the plant-associated glycoform represents a static computational model, the observed differences in SASA should not be interpreted as direct evidence of changes in Fc receptor binding, effector functions, pharmacokinetics, or immunogenicity; these potential functional consequences require experimental validation [30,31,32].

### 2.5. Pembrolizumab as a Case Study of Plant-Derived Immune Checkpoint Inhibitors

Pembrolizumab is a humanized IgG4 monoclonal antibody targeting programmed cell death protein 1 (PD-1), an immune checkpoint receptor involved in the regulation of T-cell responses. Blockade of PD-1 interactions with its ligands PD-L1 and PD-L2 can restore antitumor T-cell activity, supporting the clinical use of pembrolizumab across multiple malignancies [33]. Given the potential influence of Fc glycosylation on antibody structural and functional properties, pembrolizumab was selected as a representative immune checkpoint inhibitor to examine structural differences between experimentally resolved mammalian Fc glycans and a computationally modeled plant-associated glycoform.

Figure 4 compares the experimentally determined mammalian pembrolizumab structure (PDB ID: 5DK3) with the plant-associated GnGnXF glycoform generated as described above. Analysis focused on the conserved Fc N-glycosylation sites at Asn297 of heavy chains B and G. Importantly, the experimentally resolved mammalian glycans in 5DK3 were structurally asymmetric and were therefore analyzed individually rather than represented as identical idealized human-type glycans. The glycan associated with Asn297 of chain B lacked a resolved core fucose, whereas that associated with chain G contained a resolved fucose residue.

Panel A shows the experimentally determined mammalian pembrolizumab reference and the Fc-associated N-glycans resolved in 5DK3. The different compositions of the two experimentally resolved glycans resulted in distinct local glycosylation environments at the two Asn297 sites. Panel B shows the corresponding plant-associated model in which GnGnXF glycans containing the characteristic plant-associated β1,2-xylose and core α1,3-fucose residues were introduced at Asn297 of both heavy chains. The resulting model therefore enabled direct comparison of the plant-associated glycans with the experimentally resolved mammalian glycans while maintaining the same protein scaffold.

Panel C illustrates the surface environment surrounding the Fc-associated glycans in the mammalian reference and plant-associated model. Differences in glycan composition and spatial arrangement produced visibly distinct glycosylation environments around the Fc region. These structural representations were used to visualize the local molecular environment and were not interpreted as direct evidence of altered receptor binding or biological activity.

Panel D shows the structural superposition of the mammalian reference and plant-associated model. The corresponding Fc Cα coordinates were essentially superimposable, with an RMSD of approximately 0.000 Å. This result indicates that the Fc protein backbone was preserved during computational glycan remodeling. Because the plant-associated glycoform was generated directly from the 5DK3 protein coordinates, the near-zero RMSD primarily reflects conservation of the starting protein scaffold and should not be interpreted as evidence that glycan composition cannot affect antibody conformational dynamics under physiological conditions.

In contrast to the conserved protein backbone, substantial differences were observed in the calculated solvent accessibility of the Fc-associated glycans (Figure 4E). For Asn297 of heavy chain B, glycan SASA increased from 963.65 Å^2^ in the experimentally determined mammalian reference to 1759.94 Å^2^ in the plant-associated GnGnXF model, corresponding to an increase of 82.63%. At Asn297 of heavy chain G, SASA increased from 826.50 Å^2^ to 1440.99 Å^2^, corresponding to an increase of 74.35%. Consequently, mean glycan SASA increased from 895.08 Å^2^ in the mammalian reference to 1600.47 Å^2^ in the plant-associated model, representing an absolute increase of 705.39 Å^2^ and an overall increase of 78.81%.

The increase in glycan solvent accessibility at both Fc glycosylation sites indicates that replacement of the experimentally resolved mammalian glycans with GnGnXF structures substantially altered the modeled surface exposure of the carbohydrate moieties without altering the static Fc protein scaffold. The observation of increased SASA at both Asn297 sites, despite the structural asymmetry of the experimentally resolved mammalian glycans, further indicates that the difference was not restricted to a single Fc glycosylation site. Nevertheless, these results represent static structure-based calculations and should not be interpreted as direct evidence of changes in Fc receptor affinity, effector functions, pharmacokinetics, or immunogenicity.

Overall, the comparative analysis demonstrates that the modeled plant-associated GnGnXF glycoform of pembrolizumab retains the experimentally derived IgG4 protein architecture while exhibiting markedly different Fc glycan composition and solvent accessibility. These findings support the structural relevance of Fc glycan composition when considering plant-based production and glycoengineering of therapeutic antibodies. In this context, experimental studies of plant-produced pembrolizumab provide complementary evidence that plant-derived antibody variants can retain structural integrity, PD-1 binding activity, and PD-1/PD-L1 inhibitory function despite differences in their N-glycosylation profiles [34]. However, direct functional validation would be required to determine whether the structural differences identified in the present computational analysis translate into corresponding biological effects.

#### Functional Consequences of Fc Glycosylation and Glycoengineering in Plant-Produced Antibodies

Although structural analyses provide important information on differences between mammalian- and plant-derived antibodies, structural similarity alone does not necessarily predict biological performance. The Fc region of IgG antibodies contains a conserved N-glycosylation site at Asn297, and the composition of the associated glycan can modulate Fc conformation and accessibility, thereby influencing interactions with Fc gamma receptors (FcγRs) and downstream immune effector mechanisms. Consequently, functional characterization of plant-derived antibodies should complement structural and in silico analyses by evaluating receptor binding and biological activity [18,30,31].

Although Asn297 represents the principal and most extensively characterized N-glycosylation site in the Fc region, therapeutic monoclonal antibodies may also contain additional N-glycans, particularly within the Fab region. Fab-associated glycans can exhibit considerable heterogeneity and may influence antigen recognition, antibody conformation, and other physicochemical properties in an antibody-dependent manner. Thus, although Fab glycosylation generally has a less direct influence on FcγR-mediated effector functions than the conserved Fc glycan at Asn297, it may contribute to the functional properties of individual therapeutic antibodies [30]. In plant-produced mAbs, these additional glycans may also reflect the glycosylation capacity of the expression host, further emphasizing the importance of characterizing the overall glycan profile rather than focusing exclusively on the Fc Asn297 site [29,31].

Another relevant consideration is the stability of plant-produced therapeutic mAbs. Available experimental studies indicate that specific plant-produced antibodies and Fc-containing proteins can retain structural integrity, antigen-binding activity, and biological function after purification and under defined experimental conditions [11,12]. However, antibody stability and biological performance are highly dependent on several factors, including the antibody sequence, glycan profile, expression host, purification process, formulation, and storage conditions [30]. Therefore, rather than assuming that plant-derived mAbs are intrinsically more or less stable than their mammalian-produced counterparts, stability should be established experimentally by evaluating aggregation, fragmentation, conformational integrity, and retention of antigen-binding activity under appropriate accelerated and long-term storage conditions.

An important advance in plant molecular farming has been the development of glycoengineered plant hosts capable of reducing or eliminating plant-specific glycan residues. Conventional plant expression systems can generate N-glycans containing β1,2-xylose and core α1,3-fucose residues, which differ from the predominant glycan structures found in mammalian-produced therapeutic antibodies. To address this limitation, glycoengineering strategies have targeted endogenous β1,2-xylosyltransferase and α1,3-fucosyltransferase activities [21,24,31]. Strasser et al. generated glycoengineered *Nicotiana benthamiana* lines with reduced activity of these enzymes and demonstrated that the HIV-1 2G12 monoclonal antibody produced in these plants contained highly homogeneous N-glycans lacking detectable β1,2-xylose and core α1,3-fucose. Importantly, the resulting antibody retained antigen-binding and HIV-neutralizing activities comparable to those of CHO-derived 2G12, demonstrating that modification of plant glycosylation pathways can generate therapeutically relevant antibodies with more human-compatible glycan structures [7].

In the context of immune checkpoint inhibitors, PD-1/PD-L1 blockade represents an established therapeutic strategy in multiple malignancies [32], and the full-length structure of the human anti-PD-1 IgG4 antibody pembrolizumab has provided an important structural reference for understanding this therapeutic antibody [33]. More broadly, IgG glycosylation is an important determinant of antibody structural and functional properties, including Fc-mediated effector functions, while Fab-associated glycosylation may also contribute to antibody heterogeneity and function [34]. These considerations are particularly relevant to plant-produced immunoglobulins because plant expression systems generate distinct glycan profiles that can be deliberately modified through glycoengineering [35]. Recent experimental evidence with plant-produced pembrolizumab further demonstrates that glycoengineering can enhance FcRn binding and extend serum half-life in mice, providing direct evidence that modification of the plant-derived glycan profile can influence Fc-related pharmacological properties [36].

The relevance of these glycan modifications becomes particularly evident when considering FcγR interactions. Forthal et al. evaluated five glycoforms of the broadly neutralizing anti-HIV monoclonal antibody 2G12 produced in wild-type plants, glycoengineered plants, and CHO cells. The different glycoforms exhibited comparable binding to FcγRI, FcγRIIa, and FcγRIIb. In contrast, two glycoforms produced in glycoengineered plants lacking plant-specific β1,2-xylose and core α1,3-fucose showed significantly increased binding to FcγRIIIa compared with antibodies produced in CHO cells or wild-type plants. Furthermore, glycoforms lacking core fucose exhibited enhanced antiviral activity, demonstrating that specific alterations in Fc glycosylation can influence both Fc receptor engagement and biological activity [37].

These observations are particularly relevant to therapeutic IgG1 antibodies because FcγRIIIa is expressed predominantly by natural killer (NK) cells and plays an important role in antibody-dependent cellular cytotoxicity (ADCC). Consequently, changes in Fc glycosylation can modulate the ability of antibodies to engage immune effector cells and thereby influence their biological activity. Importantly, the relationship between glycosylation and FcγR binding is not determined simply by the presence or absence of plant-specific residues. Rather, the precise composition and structural features of Fc glycans, including fucosylation and galactosylation, can influence receptor engagement and downstream effector functions [18,30,34,37]. These findings highlight the importance of controlled glycan engineering and precise glycan characterization rather than relying solely on the removal of non-human glycan structures.

Stelter et al. (2020) [38] provided additional experimental evidence by generating distinct glycovariants of the broadly neutralizing anti-HIV antibody VRC01 in *Nicotiana benthamiana*. The authors evaluated antibodies carrying conventional plant glycans, glycans lacking α1,3-fucose and β1,2-xylose, and glycans additionally extended with terminal β1,4-galactose. Using surface plasmon resonance (SPR), they quantitatively characterized the interactions of these glycovariants with FcγRI, FcγRIIa, FcγRIIb, and FcγRIIIa. Antibodies carrying typical plant glycosylation showed limited interaction with these Fc receptors, whereas targeted glycoengineering improved and, in some cases, enhanced receptor-binding properties. These findings provide direct experimental evidence that the glycosylation profile generated by the plant host can influence FcγR recognition and that rational modification of plant glycosylation pathways can be used to modulate antibody–receptor interactions [38].

The functional consequences of glycoengineering are not limited to FcγR binding. The plant-produced lectin–Fc fusion protein Avaren-Fc provides another example in which modification of the glycosylation profile influenced biological activity. Glycoengineering of the plant expression system altered the interaction of Avaren-Fc with Fc receptors and affected its anticancer activity, illustrating that host-cell glycosylation can be an important determinant of the pharmacological properties of plant-produced Fc-containing proteins [12].

Taken together, these studies indicate that the relationship between plant-derived glycosylation and antibody function is more complex than a simple structural comparison between plant- and mammalian-derived antibodies. Conventional plant glycosylation can generate Fc glycoforms with altered receptor-binding properties, whereas glycoengineered plants can produce more controlled glycan profiles and improve the functional compatibility of plant-produced antibodies with human FcγRs [31,35,37,38]. Importantly, however, generating more human-like glycan structures should not be interpreted as automatically producing a functional profile identical to that of a mammalian-derived antibody. FcγR binding can depend on the specific glycan composition, receptor subtype, antibody subclass, and other product quality attributes [30,34,37,38].

The pharmacological consequences of antibody glycosylation may extend beyond FcγR-mediated effector functions. Glycan composition can influence the pharmacokinetic and pharmacodynamic properties of monoclonal antibodies and Fc-fusion proteins, emphasizing the importance of considering glycosylation as a critical quality attribute during therapeutic antibody development [39].

Overall, the available evidence supports a model in which plant molecular farming, glycoengineering, structural analysis, and functional characterization should be considered interconnected components of therapeutic antibody development. The transition from conventional plant expression systems to precision glycoengineering can reduce plant-specific glycan features and generate antibody glycoforms with improved compatibility with human Fc receptors. However, structural modification alone is insufficient to establish functional equivalence. Future studies should therefore integrate comprehensive glycoprofiling, FcγR-binding kinetics, cell-based effector assays, antigen-binding measurements, and in vivo efficacy studies to determine whether glycoengineered plant-based antibodies achieve functional equivalence to mammalian-produced counterparts or, in selected cases, exhibit distinct or potentially advantageous effector profiles.

The functional relevance of antibody glycosylation extends beyond structural stability, as variations in glycan composition can influence antigen recognition, immunogenicity, and Fc-mediated biological activity. Wolf et al. demonstrated that glycosylation patterns can modulate the immunological properties of therapeutic antibodies, highlighting glycosylation as an important critical quality attribute [40]. In trastuzumab, Fc glycan composition is particularly relevant to effector function; notably, afucosylated trastuzumab exhibits enhanced FcγRIIIa binding and antibody-dependent cellular cytotoxicity (ADCC), resulting in superior antitumor activity in preclinical models of HER2-amplified breast cancer [41]. These findings emphasize the importance of controlling antibody glycosylation when developing plant-based production platforms intended to generate therapeutically comparable or functionally optimized monoclonal antibodies.

## 3. Comparative Advantages of Plant and Mammalian Production Systems

Mammalian cell culture, particularly Chinese hamster ovary (CHO) cells, remains the predominant platform for the manufacture of therapeutic monoclonal antibodies because of its well-established industrial infrastructure, regulatory acceptance, process consistency, and ability to generate complex post-translational modifications compatible with human glycoproteins. These characteristics have supported the successful development and commercialization of numerous therapeutic antibodies. In contrast, plant molecular farming (PMF) has emerged as a complementary production platform with potential advantages in manufacturing flexibility, scalability, rapid production, and production costs. Techno-economic analyses have demonstrated that the economic performance of plant-based antibody manufacturing is strongly influenced by expression yield, facility scale, and downstream processing requirements [42,43]. In parallel, advances in synthetic biology are expanding the molecular engineering capabilities of plant production systems [44]. Artificial intelligence and automation are also increasingly being incorporated into bioprocess development to support process optimization, monitoring, and control [45]. Recent developments in plant molecular farming further highlight progress toward more standardized, scalable, and industrially relevant production strategies [46,47].

Mammalian production platforms generally require sophisticated bioreactors, tightly controlled culture conditions, extensive media optimization, and prolonged cell-line development and expansion processes. These requirements contribute substantially to infrastructure and manufacturing costs, particularly during the establishment and operation of large-scale facilities [43,47]. Plant-based systems, in contrast, can exploit relatively low-cost biomass production and transient expression approaches, thereby reducing certain upstream processing requirements and allowing production capacity to be adjusted according to demand [42,43,46,47]. Nevertheless, the economic advantages of PMF are not universal and depend strongly on expression levels, biomass productivity, recovery efficiency, facility scale, and downstream processing performance [42,43,47].

### Economic and Cost Considerations of Plant-Based Versus Mammalian mAb Production

The economic competitiveness of PMF depends on the integrated performance of the entire manufacturing process, including upstream cultivation and expression, biomass harvesting, extraction, clarification, purification, formulation, and quality control [42,43,47]. Unlike mammalian cell culture, in which recombinant proteins are generally produced in a liquid culture environment, whole-plant systems require recovery of the target protein from a complex biological matrix. Consequently, the economic benefits achieved during upstream production may be partially offset by the requirements of downstream processing (DSP) [42,43].

A detailed techno-economic analysis by Nandi et al. evaluated a transient *Nicotiana benthamiana* platform for monoclonal antibody production at a modeled capacity of 300 kg of purified antibody per year [42]. Using a base-case expression level of 1 g mAb/kg fresh biomass and a 65% recovery rate during DSP, the model estimated a total capital investment of approximately US$121.6 million and a cost of goods sold (COGS) of US$121/g when depreciation was included, or approximately US$90/g when depreciation was excluded [42]. Importantly, DSP accounted for approximately 65% of the capital investment and 63% of the annual operating costs in the modeled process, emphasizing that downstream operations represent a major determinant of the economic performance of plant-based antibody production [42]. These values should be interpreted as outputs of a techno-economic model rather than as commercial prices, as they depend on the assumptions, process parameters, and operating conditions incorporated into the simulation.

The same analysis showed that increasing production scale and improving antibody accumulation in plant biomass can substantially influence COGS [42]. Under favorable production conditions, plant-based systems may therefore become economically attractive for large-scale manufacturing. However, this potential economic advantage decreases when expression levels are low or when product recovery and purification are inefficient. Thus, increasing volumetric or biomass-specific productivity, improving extraction efficiency, and minimizing product losses during purification are key strategies for enhancing the economic competitiveness of PMF [42,43].

A complementary experimental cost analysis was reported by Schillberg et al. using the human M12 antibody produced in transgenic tobacco plants [43]. In this study, approximately 200 kg of leaf biomass yielded 77 g of purified antibody, with an overall recovery of 88%. The estimated total process cost was €87,550, corresponding to approximately €1137 per gram of purified antibody [43]. Notably, cultivation represented only a fraction of the total process cost, whereas DSP accounted for the largest proportion because the recombinant antibody had to be extracted from plant tissue and separated from abundant host-derived proteins and other components of the plant matrix [43]. This example illustrates why the relatively low cost of plant biomass alone does not necessarily translate into a low overall manufacturing cost.

Upstream production nevertheless represents an important advantage of transient plant expression systems. Plant cultivation does not require the large stainless-steel or single-use bioreactor capacity typically associated with mammalian cell culture, and production capacity can be scaled by increasing plant biomass rather than relying exclusively on increased reactor volume [42,43,47]. In addition, transient expression in *N. benthamiana* can generate recombinant proteins within days after gene delivery, providing greater production flexibility than the establishment and expansion of stable mammalian cell lines [43,46,47]. Recent analyses indicate that transient PMF can support rapid production and scalable manufacturing, although process consistency and integration with downstream operations remain important challenges for industrial implementation [43,46,47].

Production time represents another potential advantage of transient plant systems. Following optimization of plant growth and agroinfiltration, recombinant proteins can often be harvested within days, avoiding the prolonged development, selection, amplification, and validation processes associated with stable mammalian production platforms [43,46,47]. This characteristic may be particularly relevant for products requiring rapid deployment, including antibodies for emerging infectious diseases and rapidly evolving therapeutic pipelines. However, the overall manufacturing timeline must also account for plant cultivation, gene delivery, expression, harvesting, extraction, purification, analytical characterization, and quality-control procedures. Therefore, rapid recombinant protein expression does not necessarily translate into a proportionally shorter end-to-end manufacturing process [43,47].

Downstream processing remains one of the principal challenges of whole-plant molecular farming. After harvesting, plant biomass must be disrupted and extracted, followed by clarification and one or more purification steps to remove insoluble material, host-cell proteins, pigments, phenolic compounds, polysaccharides, nucleic acids, proteases, and other plant-derived impurities [42,43,47]. In contrast, mammalian cell culture generally produces recombinant antibodies in a relatively defined liquid medium, which can facilitate clarification and integration with established chromatographic purification processes [43,47]. Consequently, improvements in plant tissue disruption, extraction, clarification, filtration, chromatography, and overall process integration are essential for reducing product losses and manufacturing costs.

The importance of DSP is further illustrated by the M12 case study, in which four principal purification operations—Protein A chromatography, CaptoAdhere chromatography, ultrafiltration, and diafiltration—were required following the extraction and clarification of tobacco biomass [43]. Although the antibody was recovered efficiently, DSP remained the dominant cost component. These observations indicate that improving upstream expression alone may not be sufficient to achieve competitive manufacturing economics. Instead, process development should optimize the entire “plant-to-purified-product” workflow, from biomass processing through final purification [42,43,47].

Recent developments in PMF have therefore increasingly focused on integrated process design, including the optimization of expression vectors, plant cultivation, biomass harvesting, extraction, purification, and quality control [43,44,47]. Engineering strategies that increase recombinant protein accumulation, improve subcellular targeting, facilitate protein secretion, or simplify purification may reduce downstream complexity and improve overall process economics [44,47]. Moreover, the use of standardized transient expression platforms and controlled cultivation systems may improve batch-to-batch consistency and facilitate process scale-up [47].

Overall, current evidence indicates that plant-based antibody production should not be viewed simply as a less expensive replacement for mammalian cell culture. Rather, PMF represents a complementary manufacturing strategy with potential advantages in rapid production, flexible scalability, and favorable capital and operating costs under appropriate process conditions [42,43,46,47]. Mammalian systems retain important advantages in process maturity, regulatory experience, and highly standardized large-scale manufacturing, whereas plant systems may offer greater flexibility for selected products and production scenarios. The relative competitiveness of each platform ultimately depends on the specific antibody, production scale, expression level, recovery yield, purification strategy, and regulatory requirements [42,43,47].

In the future, the integration of plant molecular farming with synthetic biology, precision glycoengineering, controlled-environment cultivation, process automation, and data-driven bioprocess optimization is expected to further improve the efficiency, flexibility, and competitiveness of plant-based manufacturing platforms [44,45]. Advances in synthetic biology may enable more precise control of recombinant protein expression and product quality, while artificial intelligence and automation could facilitate process optimization, experimental design, and scale-up [44,45]. As these technologies continue to mature, plant-derived monoclonal antibodies may become an increasingly relevant component of the biopharmaceutical landscape, particularly for applications requiring flexible and scalable manufacturing [46,47]. In this context, plant molecular farming could contribute to expanding access to biologic medicines, including cancer immunotherapies, although its broader clinical and commercial impact will depend on continued progress in process standardization, regulatory acceptance, and cost-effective manufacturing [46,47].

## 4. Challenges, Regulatory Considerations, and Future Perspectives of Biofarming in Cancer Therapy

Despite substantial advances in plant molecular farming, several challenges must still be addressed before plant-produced monoclonal antibodies can achieve broader clinical and commercial adoption. Regulatory assessment represents a particularly important consideration because plant molecular farming may involve genetically modified production hosts and requires appropriate evaluation of biosafety, containment, and product-specific risks. Regulatory frameworks for molecular farming have therefore addressed both contained and open-field production of plant-made pharmaceuticals, with differences in regulatory approaches reported between jurisdictions such as Europe and the United States [48]. In parallel, advances in plant-based biopharmaceutical engineering have improved expression systems, post-translational modification strategies, and the production of complex recombinant proteins, although regulatory challenges remain relevant for the broader translation of plant-derived biopharmaceuticals [49]. Increasing automation and standardization of plant production facilities may further improve process monitoring, manufacturing consistency, and reproducibility, supporting the transition of plant-based systems toward pharmaceutical manufacturing environments [50]. More broadly, the use of genetically modified or genome-edited plants requires appropriate biosafety and environmental risk assessment within applicable regulatory frameworks [51].

Taken together, these considerations indicate that the broader implementation of plant molecular farming will depend on coordinated advances in product quality, manufacturing reproducibility, process standardization, biosafety, and regulatory development [48,49,50,51]. Regulatory assessment should consider both the characteristics of the final biopharmaceutical product and relevant aspects of the plant-based production platform, while continued technological development may facilitate more standardized and controlled manufacturing processes [48,49,50,51].

Reproducibility and process standardization represent additional challenges for the translation of plant-based antibody production into routine pharmaceutical manufacturing. Transient expression systems can enable rapid production and high levels of recombinant protein accumulation; however, variability in plant growth, infiltration efficiency, recombinant protein expression, harvesting, extraction, and purification may affect final product yield and quality [46,47,49,50]. Controlled cultivation systems, standardized production workflows, process monitoring, and improved downstream technologies are therefore important for reducing batch-to-batch variability and facilitating compliance with pharmaceutical manufacturing requirements [47,49,50].

Regulatory considerations are also closely linked to the production system itself. Depending on the host species, genetic modification strategy, cultivation environment, and intended application, plant-based manufacturing may involve regulatory requirements related to genetically modified organisms, environmental containment, product identity, purity, potency, and traceability [46,48,51]. Importantly, regulatory acceptance should not be regarded as an intrinsic limitation of plant molecular farming, but rather as an evolving process in which regulatory frameworks increasingly assess plant-derived products according to established pharmaceutical principles while also accounting for platform-specific biosafety considerations [46,48,51]. Greater harmonization of requirements and clearer regulatory pathways for plant-derived biologics could facilitate their broader translation into clinical and commercial applications [46,47,48,49,51].

Future development should therefore prioritize the integration of molecular engineering with robust process-development and quality-control strategies rather than focusing exclusively on increasing recombinant protein yield. Advances in synthetic biology, precision glycoengineering, controlled-environment cultivation, automation, and integrated bioprocessing may improve manufacturing consistency and product quality [44,45,47,49,50]. These technologies are particularly relevant to therapeutic antibodies because they can support tighter control of critical quality attributes throughout the production process.

Ultimately, the successful translation of plant-derived monoclonal antibodies into cancer therapy will depend on demonstrating reproducible product quality, appropriate pharmacokinetic and pharmacodynamic profiles, immunological safety, and clinically meaningful efficacy [40,41,46,47]. Continued clinical validation, process standardization, regulatory harmonization, and economic optimization will be essential for establishing plant molecular farming as a reliable complementary platform for the production of next-generation cancer therapeutics [42,43,46,47,48,49,50,51].

## 5. Conclusions

Cancer remains a major global health challenge, driving the development of innovative therapeutic strategies and alternative approaches for the manufacture of biopharmaceuticals. In this context, plant molecular farming has emerged as a promising platform for the production of recombinant anticancer molecules, offering potential advantages in manufacturing flexibility, scalability, and cost-effective production.

The case studies discussed in this review, particularly trastuzumab and pembrolizumab, illustrate the capacity of plant-based expression systems to produce structurally conserved therapeutic antibodies while highlighting the critical role of glycosylation in modulating their functional properties. Advances in plant glycoengineering have enabled the reduction of plant-specific N-glycan structures and the generation of more human-like glycosylation profiles, thereby improving the molecular comparability of plant-produced antibodies with mammalian-derived products and providing greater control over their potential functional properties.

Nevertheless, the broader implementation of plant-derived monoclonal antibodies in cancer therapy will depend on addressing remaining challenges related to product consistency, downstream processing, process standardization, regulatory requirements, and clinical validation. In particular, demonstrating reproducible critical quality attributes, as well as comparable pharmacokinetic, immunological, and biological properties, will be essential for evaluating the suitability of plant-based antibodies as alternatives or complementary products to conventional mammalian cell-derived biopharmaceuticals.

Future progress will likely depend on the integration of precision glycoengineering, synthetic biology, controlled-environment cultivation, automation, and advanced bioprocessing strategies. Rather than focusing solely on increasing recombinant protein yields, these approaches should aim to improve overall process robustness, product quality, and manufacturing reproducibility. Collectively, continued advances in these areas could strengthen the role of plant molecular farming as a flexible and increasingly mature platform for the development and manufacture of next-generation cancer biotherapeutics.

## Figures and Tables

**Figure 1:**
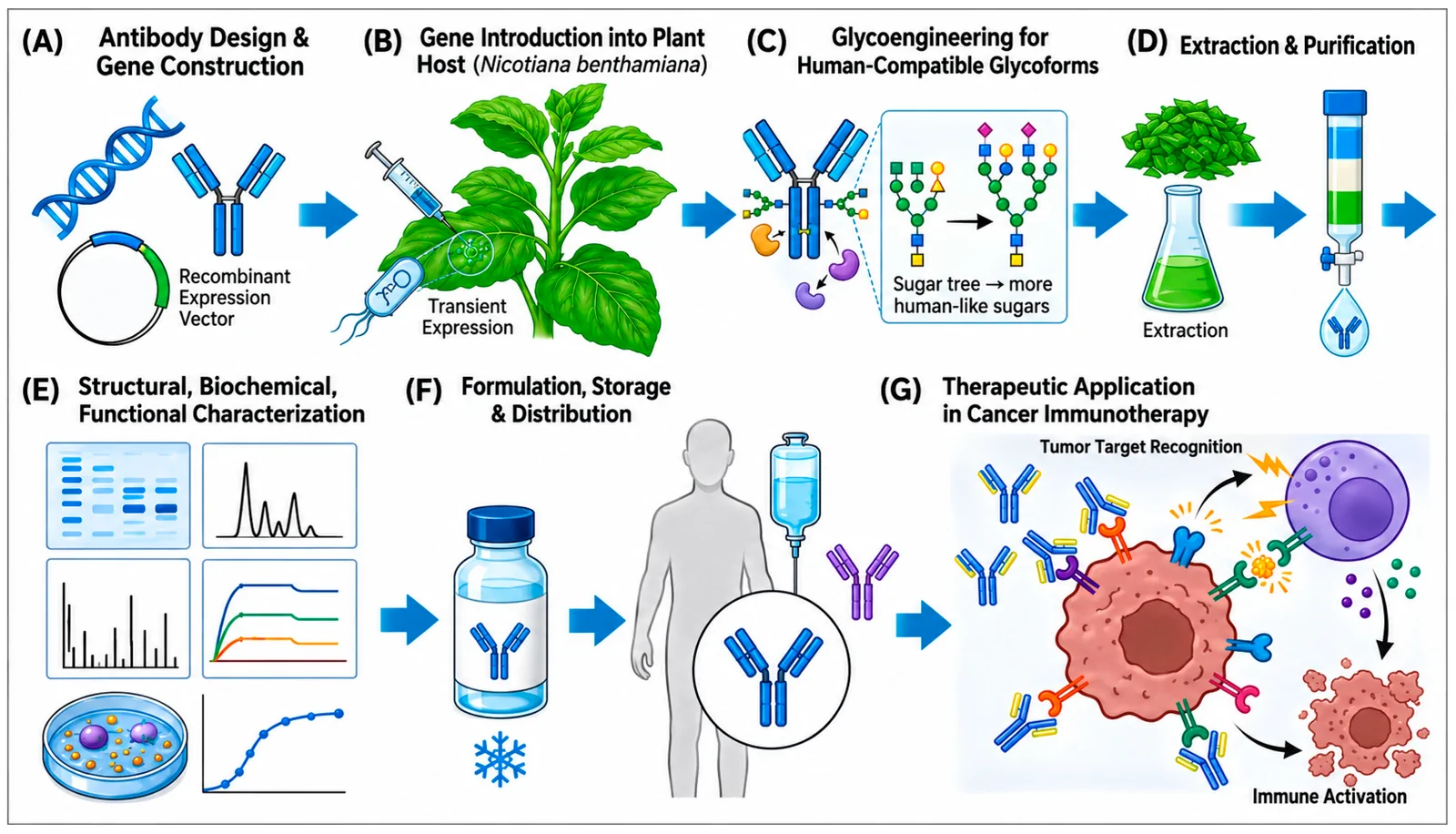
Simplified schematic representation of the plant molecular farming workflow for the production of anticancer monoclonal antibodies. The process begins with (**A**) antibody design and gene construction, including the generation of recombinant expression vectors. (**B**) The antibody genes are introduced into plant hosts, typically *Nicotiana benthamiana*, through transient expression systems. (**C**) Glycoengineering strategies are applied to modify plant glycosylation pathways and generate antibody glycoforms with glycosylation profiles more closely resembling those of mammalian-produced antibodies. (**D**) Recombinant antibodies are subsequently extracted from plant biomass and purified using downstream processing methods. (**E**) Purified antibodies undergo structural, biochemical, and functional characterization to assess product quality, integrity, and biological activity. (**F**) The final antibody product is formulated and prepared for storage and distribution. (**G**) Therapeutic antibodies are subsequently used in cancer immunotherapy, where they recognize tumor-associated targets, modulate immune responses, and contribute to tumor cell elimination. This workflow highlights the integration of plant biotechnology, glycoengineering, and biopharmaceutical manufacturing for the development of next-generation anticancer biologics.

**Figure 2:**
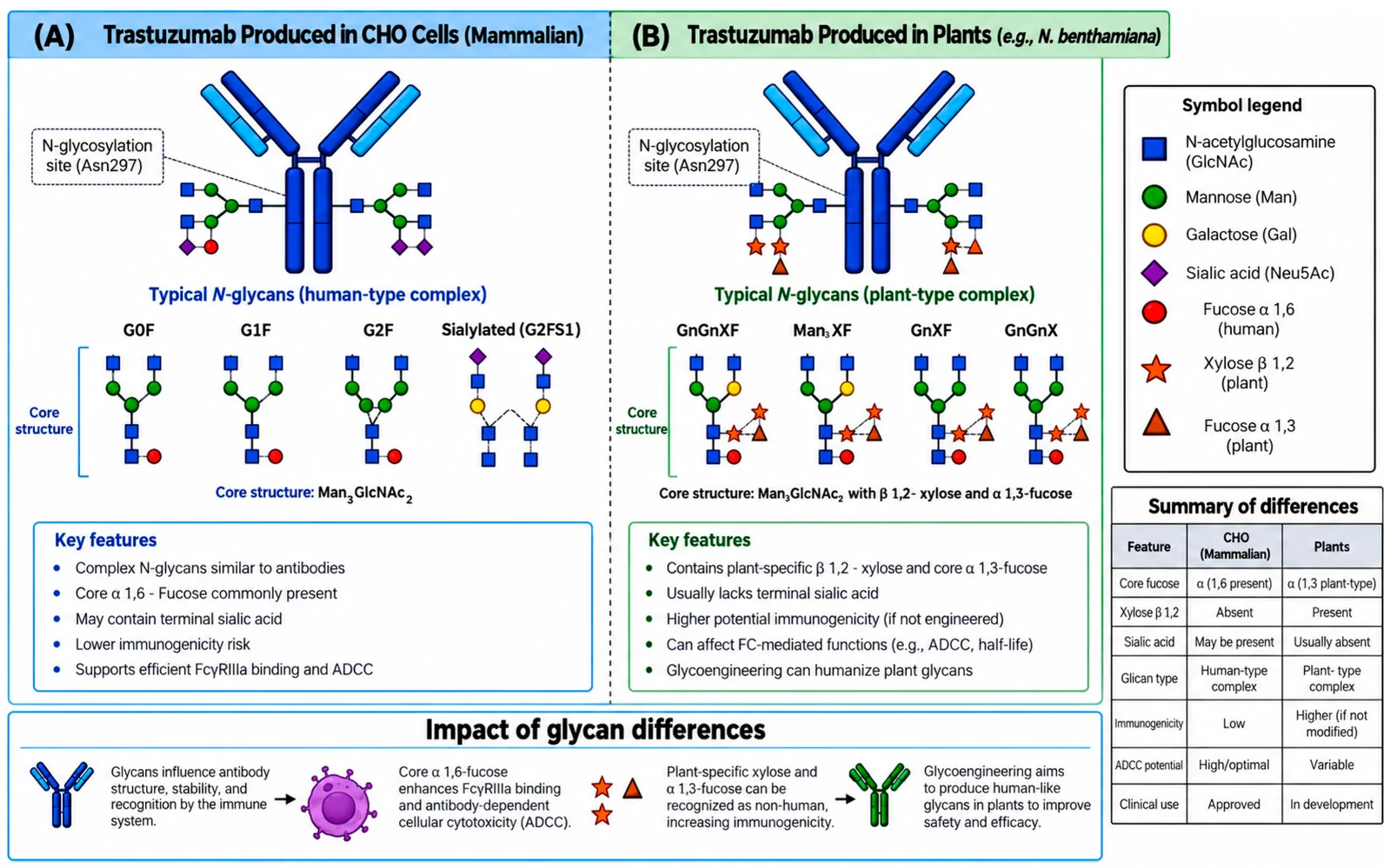
Comparative schematic representation of glycosylation differences between CHO-derived and plant-derived trastuzumab. (**A**) Trastuzumab produced in Chinese hamster ovary (CHO) cells, showing representative human-type complex Fc N-glycans associated with Asn297, including structures with core α(1,6)-fucose, terminal galactose, and sialic acid residues. (**B**) Trastuzumab produced in plants (e.g., *Nicotiana benthamiana*), showing representative plant-type complex Fc N-glycans containing plant-specific β(1,2)-xylose and core α(1,3)-fucose residues, which are not typically present in human N-glycans. These structural differences may influence Fc receptor interactions, immunogenicity, pharmacokinetic properties, and antibody-dependent cellular cytotoxicity (ADCC). Recent glycoengineering strategies aim to generate plant-produced antibodies with more human-like glycosylation profiles and improved molecular comparability for therapeutic applications [3,4,5].

**Figure 3:**
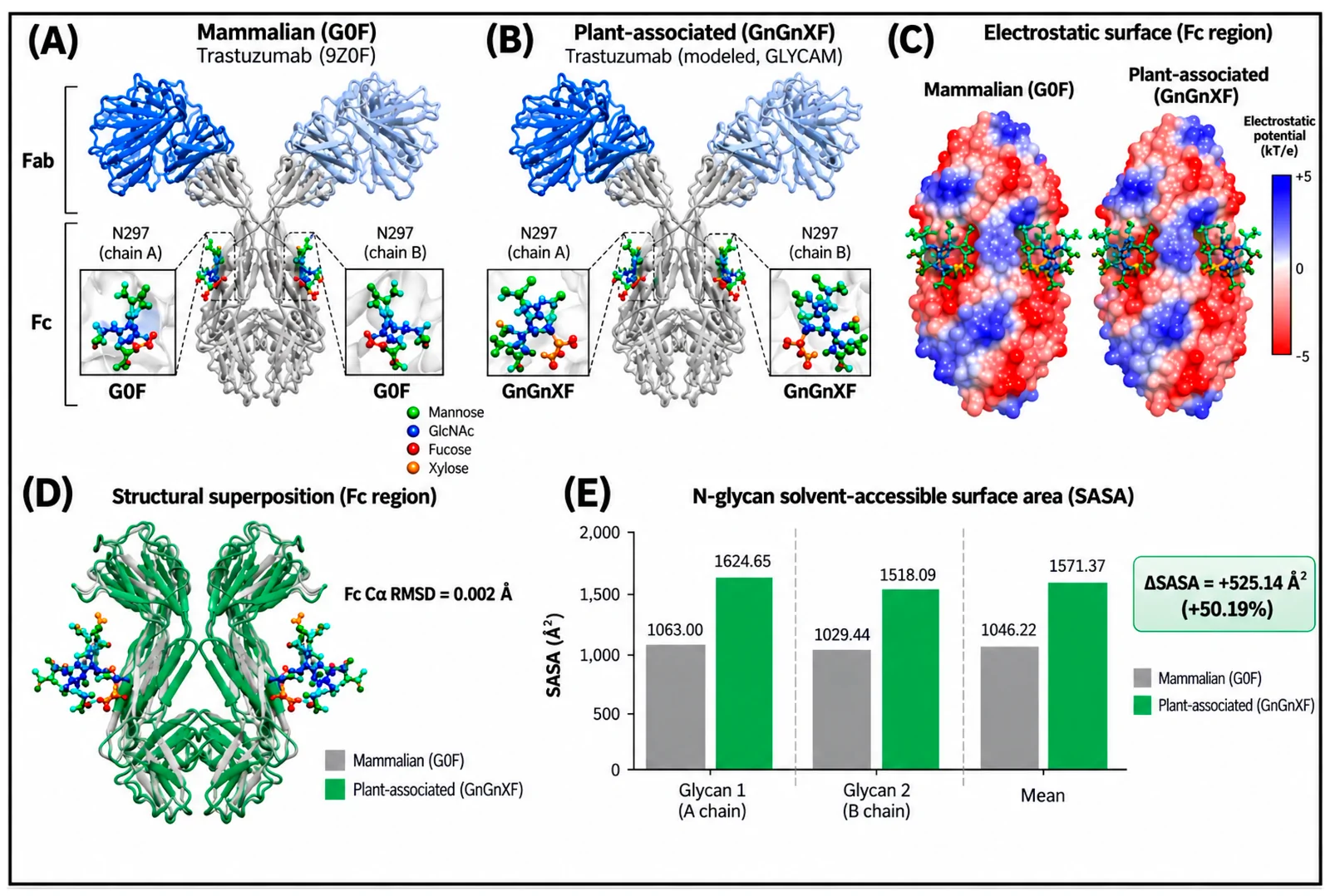
Comparative structural and glycosylation analysis of trastuzumab Fc with mammalian and plant-associated N-glycans. (**A**) Experimental trastuzumab Fc reference (PDB ID: 9Z0F) containing G0F-like N-glycans at Asn297. (**B**) Plant-associated Fc model generated from 9Z0F using GLYCAM-Web Glycoprotein Builder, with GnGnXF N-glycans containing β1,2-xylose and core α1,3-fucose at Asn297 of chains A and B. (**C**) Comparative surface representation of the Fc glycosylation environment. (**D**) Structural superposition of the mammalian reference and plant-associated models, showing preservation of the Fc backbone (Cα RMSD = 0.002 Å). (**E**) Solvent-accessible surface area (SASA) of the Fc N-glycans, calculated using a 1.4 Å probe radius. Mean glycan SASA increased from 1046.22 Å^2^ in the mammalian reference to 1571.37 Å^2^ in the plant-associated model (ΔSASA = +525.14 Å^2^; +50.19%). The plant-associated structure represents a static computational model and not an experimentally determined plant-produced antibody.

**Figure 4:**
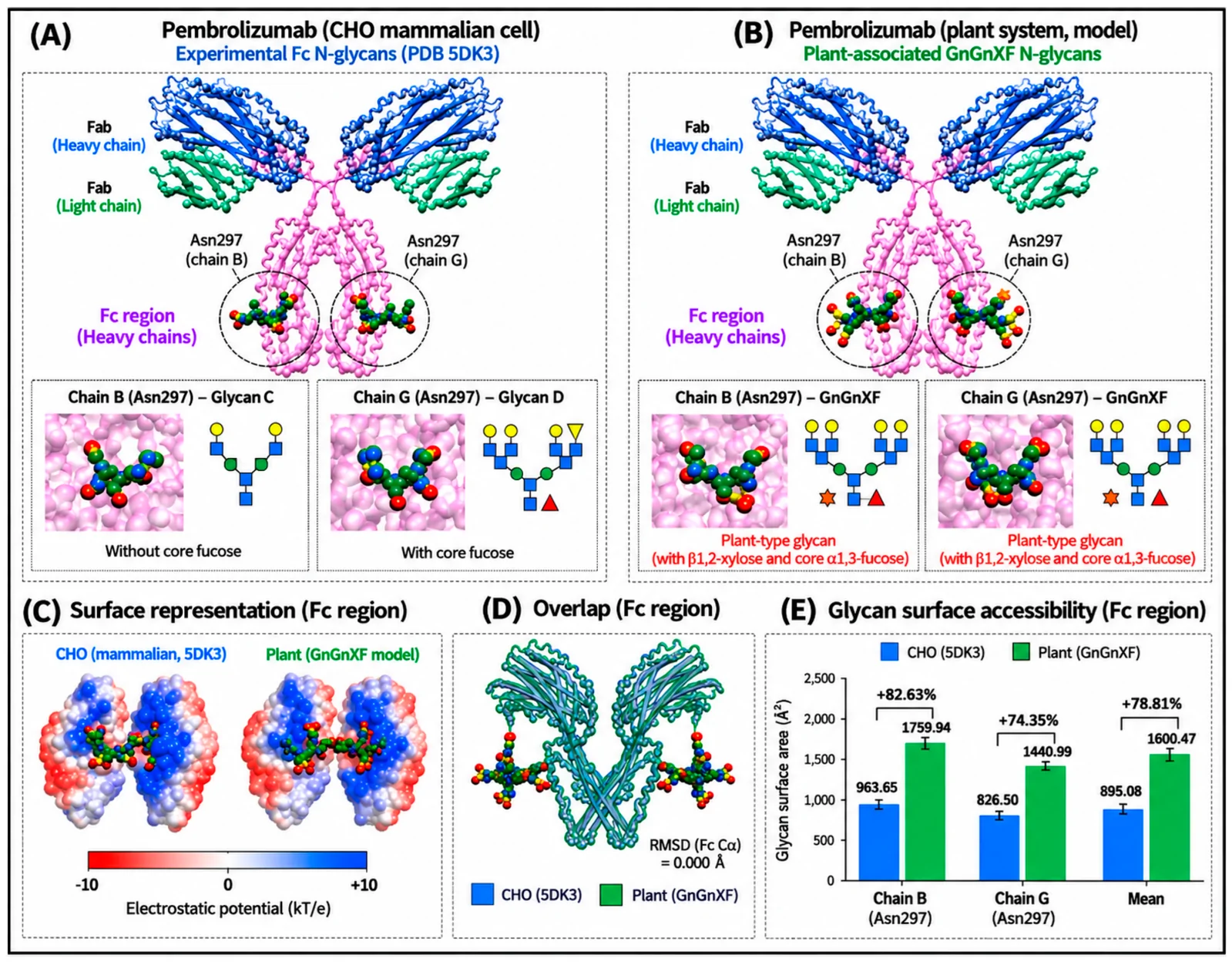
Comparative structural and glycosylation analysis of pembrolizumab with experimentally resolved mammalian and modeled plant-associated Fc N-glycans. (**A**) Experimentally determined full-length pembrolizumab structure (PDB ID: 5DK3), showing the Fc N-glycans associated with Asn297 of heavy chains B and G. The two experimentally resolved glycans are structurally asymmetric, with the B297-associated glycan lacking a resolved core fucose and the G297-associated glycan containing a resolved fucose residue. (**B**) Plant-associated pembrolizumab model generated from the 5DK3 protein coordinates using GLYCAM-Web Glycoprotein Builder, with GnGnXF N-glycans containing β1,2-xylose and core α1,3-fucose introduced at both Fc glycosylation sites. (**C**) Comparative surface representation of the Fc glycosylation environment in the mammalian reference and plant-associated model. (**D**) Structural superposition of the Fc regions showing preservation of the protein backbone during glycan remodeling (Fc Cα RMSD ≈ 0.000 Å). (**E**) Solvent-accessible surface area (SASA) ≈ of the individual Fc N-glycans. SASA increased from 963.65 to 1759.94 Å^2^ at B297 (+82.63%) and from 826.50 to 1440.99 Å^2^ at G297 (+74.35%), resulting in an increase in mean glycan SASA from 895.08 to 1600.47 Å^2^ (ΔSASA = +705.39 Å^2^; +78.81%). The plant-associated structure represents a static computational glycoform and not an experimentally determined structure of plant-produced pembrolizumab. In the structural representations, heavy-chain Fab regions are shown in blue, light-chain Fab regions in green, and the Fc region in purple/pink. Glycan atoms are represented using the molecular color scheme shown in the models, with plant-specific β1,2-xylose and core α1,3-fucose highlighted in the plant-associated glycoforms. In the electrostatic surface representation, red and blue indicate negative and positive electrostatic potential, respectively. In panels (**D**,**E**), blue denotes the CHO-derived mammalian reference and green denotes the plant-associated GnGnXF model.

**Table 1:** Representative trastuzumab formulations produced in mammalian and plant expression systems.

Commercial Name/Product	Expression System/Production Model	Main Glycosylation Characteristics
Herceptin^®^ (Genentech, South San Francisco, CA, USA)/Conventional trastuzumab	CHO mammalian cells/Stable mammalian cell culture	Core α(1,6)-fucosylated complex N-glycans with terminal galactose residues [19]
Ogivri^®^ (Biocon Biologics, Bengaluru, India), Herzuma^®^ (Celltrion Inc., Incheon, Republic of Korea), Kanjinti^®^ (Amgen Inc., Thousand Oaks, CA, USA)/Biosimilar trastuzumab	CHO mammalian cells/Recombinant CHO platform	Human-compatible Fc glycosylation profiles [23]
Experimental/Plant-derived trastuzumab	*Nicotiana benthamiana*/Transient agroinfiltration	β(1,2)-xylose and α(1,3)-fucose residues [20]
Experimental/Glycoengineered plant trastuzumab	*N. benthamiana* ΔXT/FT/Glycoengineered transient expression	Reduced plant-specific glycans and increased humanized glycans [21]
Experimental/Rice-derived trastuzumab	Transgenic rice/Stable transgenic expression	High-mannose and plant complex glycans [24]

## Data Availability

The structural data presented in this study are available in the Protein Data Bank at https://www.rcsb.org/, accession codes 6OGE, 9Z0F, and 5DK3. These data were derived from the following resources available in the public domain: Protein Data Bank (https://www.rcsb.org/).
